# The sustainable development goals in two sustainable development reserves in central amazon: achievements and challenges

**DOI:** 10.1007/s43621-021-00065-4

**Published:** 2021-12-06

**Authors:** Leonardo Capeleto de Andrade, João Paulo Borges-Pedro, Maria Cecilia Rosinski Lima Gomes, Daniel Joseph Tregidgo, Ana Claudeise Silva do Nascimento, Fernanda Pozzan Paim, Miriam Marmontel, Tabatha Benitz, Alexandre Pucci Hercos, João Valsecchi do Amaral

**Affiliations:** Mamirauá Sustainable Development Institute, Estrada Do Bexiga, Tefé, AM 258469553-225 Brazil

**Keywords:** River dwellers, Riverine communities, 2030 Agenda, Global Goals, Amazonia

## Abstract

The 2030 Agenda was set in 2015 by the United Nations, with 17 Sustainable Development Goals. The Amazonian riverine people are recognized as traditional communities that have their own culture and use the local natural resources of their territories in an ancestral and traditional way. The Sustainable Development Reserve is a Brazilian protected area category which aims to ensure the protection of the natural environment while allowing the residence and the use of these lands by traditional populations. This article reports and discusses the achievements and challenges of the Sustainable Development Goals in two sustainable development reserves in Central Amazonia. The goals were evaluated in the Mamirauá and Amanã Sustainable Development Reserves, due to the large research programs developed in those areas along the past 20 years. The 17 Sustainable Development Goals have a clear connection with the mission of these sustainable development reserves in Central Amazon. Despite the many achievements conquered over the years, there are many challenges yet to overcome; and while striving to achieve the goals from the 2030 Agenda, new challenges will emerge. The current main challenges to reach the Sustainable Development Goals in the Mamirauá and Amanã Sustainable Development Reserves, in Central Amazon, are connecting to the reality of rural areas.

## Introduction

**Fig.1:**
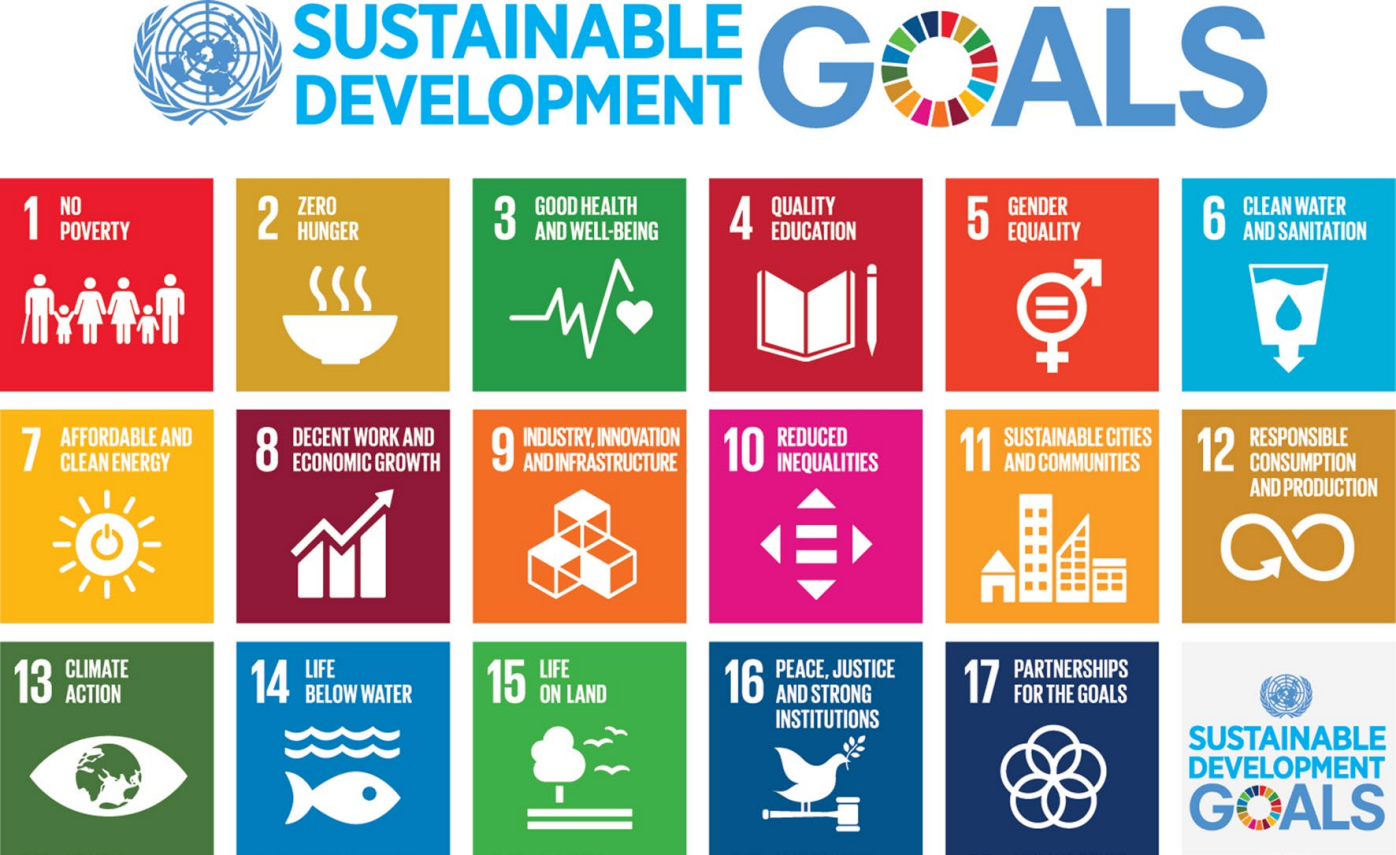
The Sustainable Development Goals of the 2030 Agenda of United Nations. Source: United Nations (2015)

A long-term "*global agenda for change*" was proposed by the United Nations in 1987, to achieve sustainable development. Sustainable development is "*development that meets the needs of the present without compromising the ability of future generations to meet their own needs*"; requiring "*meeting the basic needs of all and extending to all the opportunity to fulfill their aspirations for a better life*" (WCED, 1987) [1]. In 2015 the "2030 Agenda" was set, including 17 Sustainable Development Goals (SDGs) and 169 targets to be achieved by 2030 (Fig. 1)—in areas of crucial importance to humanity and the planet [2].

The Amazon River watershed encompasses almost seven million km^2^, carrying about 1/6 of all the water that flows to the oceans. Through this rainforest there are rivers, streams, canals, lakes, marshes, sandy beaches, floodplains [3, 4]—and the forest people, as the river dwellers.

The riverine people (*ribeirinhos*) are the people who live along the rivers, as many do in the Central Amazonia. In Brazil, they are recognized as traditional communities that have their own culture and use the local natural resources of their territories in an ancestral and traditional way [5]. These river dwellers largely descend from native people (indigenous) and rubber tappers [6], usually live in stilt-houses (*palafitas*) or floating-houses (built over logs)—most constructed with native woods extracted in the region. The main economic activities of these communities involve fishing, small-scale farming, extractivism (timber, açaí fruit *Euterpe oleracea*, Brazil nuts *Bertholletia excelsa*), handicrafts, local commerce and community-based tourism [7].

The riverine communities of Central Amazonia exist in a challenging environment for human development due to the annual flood pulses, waterway-only transport, long distances from urban centers, low levels of formal education and a lack of public energy supply and infrastructure. On the other hand, the rainforest has a large natural amount of food, and a highly adapted traditional people with an ancient knowledge about the Amazon. The rainforest has a high biodiversity of plants and animals that provide many food sources to the forest people; moreover, local community production (such as fish and manioc flour) provides subsistence.

In the Brazilian Amazon there are 346 Protected Areas (PA), covering 28% of the territory [8]. The Sustainable Development Reserve (SDR) is a Brazilian PA category which aims to ensure the protection of the natural environment while allowing the residence and the use of its lands by traditional populations, with sustainable systems of exploitation and natural resource management [9]. The first declared SDR was Mamirauá (1996), followed by Amanã (1998); both in the Mid-Solimões region, in Central Amazonia [10, 11].

Many programs and projects have been implemented with the riverine people living in the Sustainable Development Reserves in the Central Amazonia. This case of study aims to report and discuss the achievements and challenges of the Sustainable Development Goals in the first two Sustainable Development Reserves: Mamirauá and Amanã.

## Material and methods

**Fig.2:**
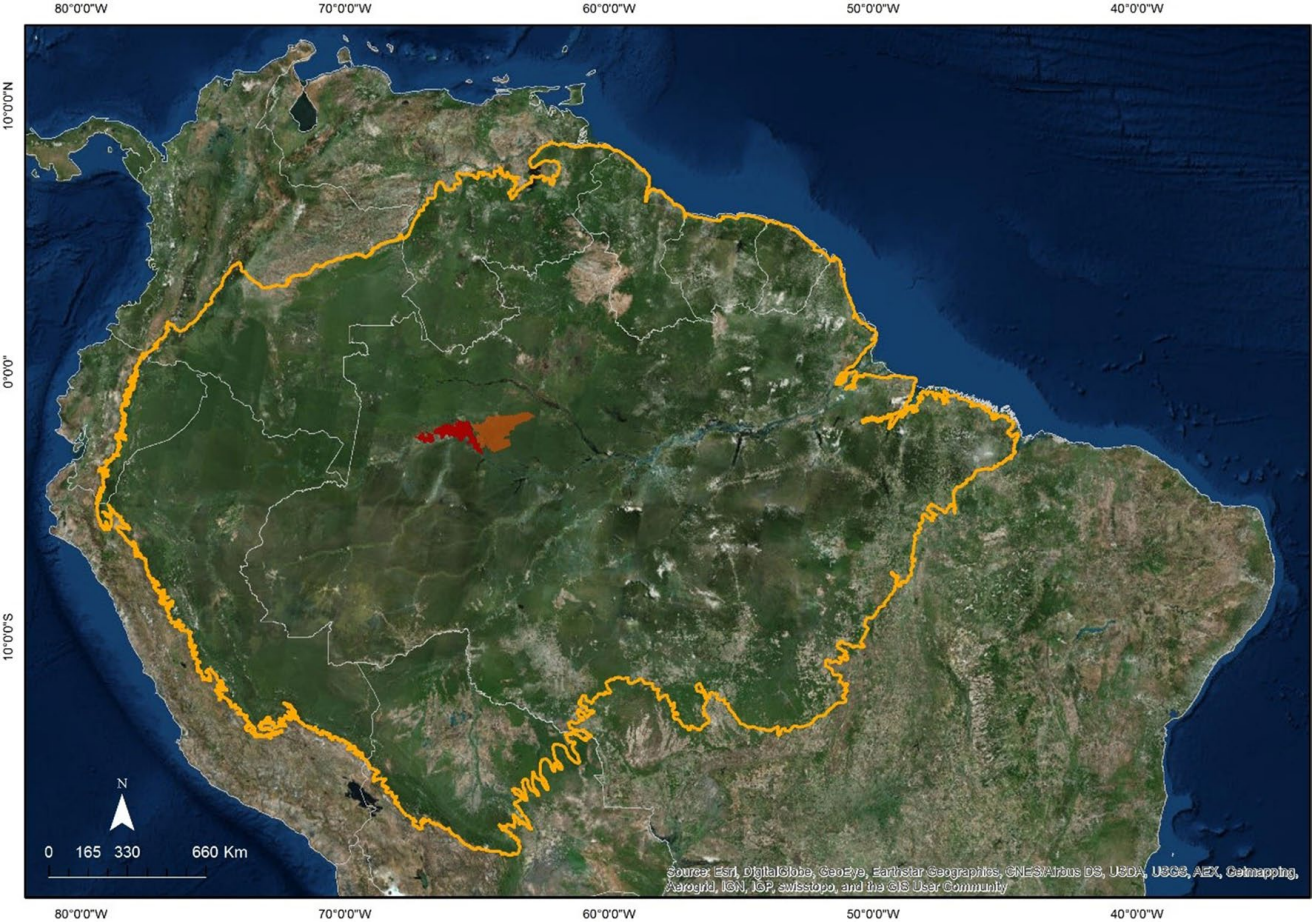
Location of Sustainable Development Reserves Mamirauá (in red) and Amanã (in orange), in the Central Amazon, Brazil

The goals were evaluated in the two contiguous Sustainable Development Reserves (SDR), Mamirauá and Amanã (Fig. 2). The Mamirauá SDR was established in 1996, being the first of its category [10]; the Amanã SDR was created in 1998 [11]. In addition to being the first two of its kind, the Mamirauá and Amanã SDR were used as cases due to the large research programs developed in those areas over the last 20 years.

Mamirauá SDR is entirely formed by floodplain areas (*várzea*), while Amanã SDR mostly encompasses non-floodable upland areas (*terra firme*). Together, they cover 34,740 km^2^, with 337 riverine communities and 16,339 inhabitants; some of these communities have less than five houses, others more than 100 [7, 12]. These riverine communities belong to the cities of Alvarães, Uarini, Fonte Boa, Jutaí and Maraã—Amazonas state, Brazil.

The 17 Sustainable Development Goals [2] were qualitatively evaluated on a local level according to the reality of the Central Amazon’s reserves. Targets related to other regions, biomes or realities (e.g. desertification) were not considered. The three most related targets were mentioned in the opening of each goal; the cases connected to each of the goals and targets were cited; and the most challenging targets were highlighted last. The information was researched from books, articles and reports through keywords “Sustainable Development Reserve” (in Portuguese and English), plus “Mamirauá” and/or “Amanã”.

## Results and discussion

### SDG 1: No poverty

Goal 1 aims to “End poverty in all its forms everywhere”, with targets such as: (1.1) eradicate extreme poverty; (1.3) implement social protection systems; and (1.4) equal rights and access to economic resources, basic services and technology [2]. Poverty is more than the lack of income and resources to ensure a sustainable livelihood. The economic growth, as the social protection systems, must be inclusive to provide sustainable jobs and promote equality [2].

The households in the riverine communities of Mamirauá and Amanã SDR have an average of six members and an average annual income around US$ 4600–5450—45% of which is derived from government benefits, 37% from family production, and 17% from payments of wages and services [13, 14]. Despite the low family income, the monthly values are higher than the Brazilian national minimum wage, and also higher than the average from the municipalities in the region [7, 11, 12].

The management projects implemented in the Mamirauá and Amanã SDR (such as the Fisheries Management and Community-Based Tourism) have increased incomes over the years (Table 1). Areas where *pirarucu* (*Arapaima gigas*) fishing management projects were implemented increased the average income of families by 15% (MSDI, 2011). The financial stability of families is represented by the purchase of domestic goods, which are usually bought in cash in the region’s towns. The tail motor (*rabeta*) is the most common domestic item in the communities, with usually more than one per household; followed by a gas stove, television, bed, and cell phone—present in more than 65% of households [11].

**Table 1:**
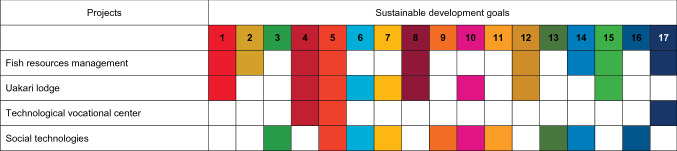
Projects developed in the Mamirauá and Amanã Sustainable Development Reserves (SDR) and their connections with the Sustainable Development Goals (SDGs)

Despite the low income, the biggest challenge to end poverty and vulnerability in rural Amazonia is to ensure that social policies and protection systems (*Targets 1.3, 1.4, and 1.5*) fully reach the region, as the basic services—*Health* (*SDG 3*), *Education* (*SDG 4*), *Water and Sanitation* (*SDG 6*), *Clean Energy* (*SDG 7*), communication, social security and income distribution, and access to land and use of natural resources for family maintenance. These are determining factors for reducing inequalities and poverty.

### SDG 2: Zero hunger

Goal 2 aims to "End hunger, achieve food security and improved nutrition and promote sustainable agriculture", with targets such as: (2.1) ensure access to safe, nutritious and sufficient food all year round; (2.2) end all forms of malnutrition; and (2.4) increase sustainable food productivity [2]. Although Brazil has left the “Hunger Map” and hunger is not the main problem for the local riverine population, food security issues are critical in Central Amazonia. The Brazilian protected areas, including the SDR, aim to protect the natural resources necessary for the livelihood of traditional populations [9].

Manioc is the main source of carbohydrates for the river dwellers and other traditional Amazonian peoples; most of the manioc is planted for artisanal manioc flour production, which is an important cultural part of the local diet [15]. However, in the floodplains the harvesting period is controlled by the water level; during the flood period it is not possible to plant as the water inundates the fields, and fishing becomes harder [16].

The management of *pirarucu* increased the fish productivity (from 3 to 650 tons per year, between 1999 and 2017) also in addition to the natural stocks in the region [17]. Yet, the positive impacts of this management cannot be measured by a single indicator: the protection of the lakes also benefits many fish species, including smaller species with great food importance to the local communities.

Wild meat is other important protein source for rural Amazonian populations; however, uncontrolled hunting may lead to a decrease in populations of some species—such as the paca *Cuniculus paca* [18]. Thus, it is necessary to quantify the target fauna [19] and establish the management of the main hunted species [20]. The regulation and management of subsistence hunting are imperative to ensure food security to the forest inhabitants and the wildlife resources on which they depend.

Increasing incomes and better access to markets has improved the standard of living of riverine people; however, this also induced changes in dietary patterns, such as the increase in the consumption of commercial chicken and industrialized food over fish or other regional products—impacting the health of those communities. Thus, the main challenge to improve the food security in this region is to avoid malnutrition (*Target 2.2*) through the incentivization of small-scale sustainable food production (*2.3 and 2.4*).

### SDG 3: Good health and well-being

Goal 3 aims to "Ensure healthy lives and promote well-being for all at all ages", with targets such as: (3.2) end preventable deaths under five years of age; (3.3) end epidemics and neglected tropical diseases; and (3.7) universal access to sexual and reproductive care, family planning and education [2].

Infant mortality is an indicator related to educational, health, and sanitation policies; while this rate in the region of Mamirauá and Amanã SDR was almost twice the Brazilian figure in 1991, the values equaled it in 2010 [12]. In 1991 the infant mortality rate was 86 per 1000 live births; this rate dropped to 28 per 1000 in 2011 [12]. In 2014 this rate reached 15 per 1000 in some municipalities of the region [21]; and in 2018, was further reduced to 14 per 1000 live births—being for the first time lower than the Amazonas State rate, 17 per 1000 [11]. Together with the reduction in infant mortality there was also a reduction in the number of children per woman; from an average of 10 in 2001 [22] to five children per woman in 2018 [11]. Those improvements were a result of the continuing health-care education, such as adequate breastfeeding, immunization, and other sexual and reproductive information [12], by working with traditional midwives in the region.

A vast diversity of biological species is found in the Central Amazon; many of them are vectors of pathogens or microbial species and parasites associated with human diseases—such as malaria, leishmaniasis, schistosomiasis, helminthiasis, and Chagas disease. Deforestation and land use and environmental changes affect the dynamics of these diseases [23]. Those diseases occur in combination with a large prevalence of gastrointestinal disorders in the region [21]. Each dollar invested in water and sanitation saves 4.3 dollars in public health; specifically in Brazil, this cost–benefit ratio is 8.93 for investments in sanitation and 2.5 for water [24]. Thus, *Clean Water and Sanitation* (*SDG 6*) improvements help to avoid associated health problems [25].

The low level of education (illiteracy), the common presence of poisonous animals, the long-distance from urban centers, and the natural conditions of the forest also aggravate the good health and well-being solutions to the riverine people [21]. Thus, the main challenge is to combat especially the water-borne and other neglected tropical diseases (*Target 3.3*) that are so common and associated with this region.

### SDG 4: Quality education

Goal 4 aims to "Ensure inclusive and equitable quality education and promote lifelong learning opportunities for all", with targets such as: (4.3) equal access to affordable technical, vocational and higher education; (4.4) increase in the number of people with relevant skills for employment; and (4.6) universal literacy and numeracy [2].

In rural Amazonia many children have difficulty accessing formal schools, usually having classes in mixed-age class primary schools in or near their communities. To attend high school, many students need to travel or move to the towns and cities in the region [12]. The decrease in the high number of children per woman over the years [11] also influenced the increase in the number of years of education of these young women, who do not need to drop out of school due to pregnancy.

In the last 20 years many river dwellers received training to improve employment skills, such as the case of Uakari Lodge—a Community-Based Tourism (CBT) project in the Mamirauá SDR [26]. In the lodge locals provide services as managers, local guides, boat drivers, janitors, maintenance workers, waiters, cleaners, and cooks. Thus, residents from the communities in the region that work in the Uakari Lodge receive training to improve their experience with the guests (including many foreigners), such as English language skills; food and beverage preparation; birdwatching and photography skills; and solar energy maintenance [27].

Technological Vocational Centers (CVT) are Brazilian educational units focused on the dissemination and transfer of scientific and technological knowledge—currently with more than 400 operating in Brazil [28]. The “Social Technologies of Amazonian Floodplain” CVT was created in 2013, in the Central Amazon, and aims to train young people on the sustainable management of natural resources in the protected areas, and to implement improvements in the quality of life of the residents of their traditional communities (including the river dwellers). The candidate students are nominated by their communities or organizations, receiving a scholarship for their maintenance in the town during their studies—and returning to their communities by the end of the program to implement their projects (Table 1).

The major challenge in this rural region is to reach universal primary and secondary (and especially university) education to all the people (*Targets 4.1, 4.2, 4.3*), partly due to the logistic difficulty in this rainforest region. Promoting quality education in the rural areas is a world challenge.

### SDG 5: Gender equality

Goal 5 aims to "Achieve gender equality and empower all women and girls", with targets such as: (5.1) end all forms of discrimination; (5.5) ensure women’s full and effective participation and equal opportunities for leadership; and (5.A) give women equal rights to economic resources [2].

The typical gender division of household labor has a sexual asymmetry in rural Central Amazonia; hunting and fishing are traditionally a responsibility of men, whilst house and child care are of women—both participating in agricultural activities. Thus, in the past women had a limited labor market insertion and poor access to money, in a position of subordination and dependence on men [29].

The Fisheries Management is an artisanal, family-centered, and sustainable activity that has been historically associated with male labor in the Central Amazon. Nowadays women have autonomy and protagonism in all stages of this fishing activity, as well as in the Associations; the gender equity is currently registered in the Internal Rules of the Arapaima Management Project [30]. In 1999 there was no formal participation of women in these fishing activities; in 2017 women were active in 10 of the 12 projects, representing 38% of beneficiaries [17].

The ecotourism in Mamirauá SDR (as the Uakari Lodge) also increased household and women's income (with trade and labor) and consequent women’s economic independence, autonomy, and empowerment (Table 1). Although initially playing typical social roles (such as cleaning and cooking), within a few years women were acting as tour guides and managers [29].

Handicrafts are traditionally produced and marketed by women at Amanã SDR. In addition to an economic alternative, the organization and strengthening of these groups of women artisans is a political project to empower and build a regional, social and environmental identity and visibility of traditional lifestyles—with women as protagonists [31].

The women in the region are increasingly participating and leading labor associations and studying longer to look for new opportunities. Nevertheless, despite the advances in women's empowerment in those activities, there are still barriers with the routine, home and childcare activities (*Target 5.4*)—largely associated with women's work in these regions for patriarchal reasons [30]. Eliminating all forms of discrimination (*Target 5.1*) and ensuring equal opportunities for leadership (*5.5*) are constant challenges.

### SDG 6: Clean water and sanitation

Goal 6 aims to "Ensure availability and sustainable management of water and sanitation for all", with targets such as: (6.1) safe and affordable drinking water; (6.2) achieve access to sanitation and hygiene and end open defecation; and (6.B) support and strengthen the participation of local communities in improving water and sanitation management [2].

Paradoxically, Amazonian populations live in a region with water abundance, but have difficulty accessing potable water [12]. Until the early 2000s, over 90% of people in riverine communities had no piped water, transporting it from the nearest river to their homes in buckets or bottles [32].

A water supply system implemented in many riverine communities of the Central Amazon floodplain (e.g. Mamirauá SDR) has a photovoltaic pump over a floating wooden raft and a water tank on a high timber stand; rainwater is also commonly collected and used in these regions. Community residents participate in the entire assembly process (including the construction of towers and rafts with local timber) and in the maintenance of the systems, appropriating the technologies [32]. Through this system it was possible to bring piped water into households, reducing physical effort (especially for women, who traditionally performed such work). Water supply and sanitation technologies even influence the reduction of rural exodus in these regions [6, 33].

Open defecation (or untreated) is still common and cultural in riverine communities in Central Amazonia. Pit latrines are the most common type of sanitation found in these rural communities—being underwater in the flood seasons; experimental dry toilets (two-chamber) were implemented in some communities, but the technology was not appropriated by these populations [33, 34].

Despite all efforts, there is still a lack of adequate water supply and sanitation solutions for the riverine people of Central Amazonia [32]. The flood-pulse of the floodplain creates complex conditions for adequate access to water treatment and sanitation solutions (*Targets 6.1, 6.2, and 6.3*); the absence of electricity also hampers the implementation of some technologies. Point-of-Use solutions to household water treatment are encouraged to compensate the deficiencies in the system. However, to end open defecation (*Target 6.2*) it is necessary to improve public policies, sensitizing residents, and developing adapted technology.

### SDG 7: Affordable and clean energy

Goal 7 aims to "Ensure access to affordable, reliable, sustainable and modern energy for all ", with targets such as: (7.1) universal access to modern energy; (7.2) increase in global renewable energy; and (7.A) promote access to research, technology and investments in clean energy [2].

The traditional source of electricity in many rural communities of the Amazon is generators (fueled by diesel or gasoline), usually limited to an average of four hours of use per day. Conventional distribution systems in the Central Amazon are impeded due to the environment, the remoteness of areas, and the low population density [35]. Thus, solar energy has been recommended for this tropical region as early as in 1998 due the benefits outweighing the cost of installation [32].

Even with power limitations, home solar systems improve the standard of living of riverine people—enabling the use of lighting, television, radio, and reducing the use of candles and kerosene lamps. Solar energy also improved schools and allowed access to the internet in some communities [35], beyond the photovoltaic pump used in the water supply [32].

Nowadays, the riverine communities of Central Amazon are using photovoltaic panels as a power source in homes, schools, soccer field lighting, community radios, fruit pulp processing, and ice machines (MSDI 2019*b*). The current main challenge is to disseminate these technologies (*Targets 7.1, 7.2, and 7.3*), stimulating the local commercialization of their components (focused on the main household uses and with appropriate cost for these populations)—bringing light to all and replacing traditional generators.

### SDG 8: Decent work and economic growth

Goal 8 aims to "Promote sustained, inclusive and sustainable economic growth, full and productive employment and decent work for all", with targets such as: (8.2) economic productivity through diversification, technological upgrading and innovation; (8.7) eradicate forced and child labor and modern slavery; and (8.9) promote sustainable tourism that creates jobs and promotes local culture and products [2].

The debt bondage was very common in the Central Amazon, especially during the "Rubber Boom". This system was a closed economy of debts and barter—in a relationship of dependence and subordination. The *patrons* (bosses) were the only links with the market, profiting on both ends [36]. Nowadays the inhabitants of the Central Amazon have freedom of trade, buying and selling their products in the nearby towns and cities.

Fishing is one of the most traditional economic activities in the riverine communities in Central Amazonia (as in Mamirauá SDR). The management of *pirarucu* generated revenue increases from US$ 6000 in 1999 to US$ 850,000 in 2017; and the number of fishers started from 41 in one area, to 1590 in 12 areas, 43 communities, three fishing colonies, and one association in the same period. During this period 5,000 tons of *pirarucu* were produced, reaching the national market [17].

Community-Based Tourism (CBT) supports the economy by employing people in their local livelihoods—benefiting their quality of life, enhancing their culture, and contributing to the development and the sustainability of their communities and their environment [37]. The Uakari Lodge promotes sustainable ecotourism in the Mamirauá SDR (Table 1), with the direct involvement of local communities in the activities, as well as providing services and selling local products [26]. This CBT case increased the income from around US$ 1500 in 1998 to around US$ 90,000 in 2011—and from 17 to 81 families involved in the same period (MSDI, 2011).

The recognition of geographical indication and designation of origin of products, such as the *Uarini* manioc flour [38], adds value to traditional local products. The future designation of origin of *pirarucu* from Mamirauá SDR will increase value of the product and open new markets as well. Also, the organization and valorization of local handicrafts expands the markets and the income generation to the traditional artisans [31].

The continuous protection of the Central Amazon rainforest is of a paramount importance, as it represents the sustainability of tourist attractions, socio-cultural resources, employment and the quality of life of its residents. The current challenge is to maintain this sustainable economic growth (*Targets 8.1 and 8.2*) to improve the quality of life of these forest people.

### SDG 9: Industry, innovation and infrastructure

Goal 9 aims to "Build resilient infrastructure, promote inclusive and sustainable industrialization and foster innovation", with targets such as: (9.1) develop sustainable infrastructure; (9.2) promote inclusive and sustainable industrialization; and (9.C) increase access to information and communications technology [2]. Beyond the difficulties to infrastructure and industrialization that occur in rural areas, the Amazon exacerbates these problems, with a giant territory that challenges the logistic of the production chain. Products in Central Amazonia are commonly marketed *in natura* (such as fish, fruits, and wood) or processed with low-tech and without standardization—such as natural oils [39]. Such production usually lacks adequate infrastructure support, as with the *pirarucu* fish [40] and manioc [41].

Quality control of fish production has been the target of several institutions, strengthening the supply chains to small scale industrialization and market access. This led to the development of suitable solar-powered infrastructure for processing, and good practices [42] such as ice production for fish preservation [43], and product traceability (*Origens Brasil*). To facilitate wildlife exploitation (fish and caiman), floating processing structures have been developed in the Mamirauá SDR, adapted to the reality of the local setting by using solar energy and treated rainwater.

The definition of good practices in the process of extracting oil from andiroba *Carapa guianensis* seeds [44], and the development of equipment for this purpose are also advances to reduce processing time and improve product quality. Similarly, a fruit pulp processing plant with a solar cooling system was installed at Amanã SDR, producing more than a ton of fruit pulp in 2018.

Despite the advancements, challenges remain in the absence of consolidated adequate infrastructure—such as electricity, communication, and logistics (*Targets 9.1, 9.2, and 9.C*)—in the development of a business social-oriented sector, in the access to better markets for processed products, in the competition with products of illegal or non-sustainable origins, and in the slow and complex licensing processes.

### SDG 10: Reduced inequality

Goal 10 aims to "Reduce inequality within and among countries", with targets such as: (10.2) empower and promote the social, economic and political inclusion; (10.3) ensure equal opportunities and end discrimination; and (10.B) encourage official development assistance and financial flows [2].

As previously mentioned, Brazilian SDR aim to promote biodiversity conservation, while ensuring the conditions for improving the quality of life of traditional populations [9]. Many projects raised national and international funds to improve the health, welfare, and income of the Amazonian riverine people that live in reserves, such as the Mamirauá and Amanã SDR.

Currently, each community from Mamirauá and Amanã SDR has an organizational structure and political associations for decision making [45]. These organized groups build and implement their own rules to occupation, management, division of extraction quotas and profit, or even the application of penalties. Profits from CBT in the SDR are shared among the involved communities—considering the participation, ecotourism activities, and the rules of natural resources [46].

In the fisheries management initiatives, the sharing of the quotas is carried out by fishers' associations and obey criteria collectively defined in assembly meetings. Initially, the quota is divided equally among the participants; however, those who performed the most activities on behalf of the system (accountants, management, wardens) earn an additional percentage, and those who breached the rules are penalized by reducing their quotas [47].

Over the years, civil society organizations have helped associations to gain access to public policies, such as closed season insurance and the definition of minimum prices for fishing. Despite the achievements, many challenges still exist in economic (*Target 10.1*) and political (*10.4*) inclusion realms in this region.

### SDG 11: Sustainable cities and communities

Goal 11 aims to "Make cities and human settlements inclusive, safe, resilient and sustainable", with targets such as: (11.1) ensure access to adequate, safe and affordable housing and basic services; (11.4) strengthen efforts to protect and safeguard the natural heritage; and (11.C) support sustainable and resilient buildings utilizing local materials [2].

The riverine people typically live in wooden houses near the rivers, in communities usually without roads connecting them. In the floodplain these residences are stilt-houses or floating-houses commonly built by the inhabitants themselves with regional wood, in a sustainable way; the traditional thatched roofs have been replaced with corrugated fiber cement or aluminum sheets over time, because they are safer (avoiding animals and insects) and more durable [12].

Until few years ago, those houses had no power, no water supply system, and no sanitation improvements; energy was limited to generators, water was taken directly from rivers, and open defecation was a common practice. Currently, domestic solar systems (*SDG 7*), as well as water and sanitation facilities (*SDG 6*), are becoming more common in Central Amazonian communities [12, 32, 34].

Despite the typical sustainable lifestyle of the riverine people, the biggest challenge in this region is the distance from the urban centers (*Target 11.2 and 11.6*)—making the access to some technologies (such as solar panels) or materials (to water supply and sanitation systems) difficult. The transport in the rural areas of Central Amazon is limited to boats; so, it increases the prices of those non-local materials and hinders a quickly spread throughout the communities. Also, the dissemination of the tested technologies in this region is still a constant challenge.

### SDG 12: Responsible consumption and production

Goal 12 aims to "Ensure sustainable consumption and production patterns", with targets such as: (12.2) achieve the sustainable management and efficient use of natural resources; (12.5) reduce waste generation; and (12.A) support scientific and technological capacity for sustainable consumption and production [2].

To this day manioc flour and fish remain the staple diet of Amazonia’s riverine population [48], and are the main sources of carbohydrates and protein respectively. Moreover, wild meat is also important, particularly during fishing lean seasons [16, 49]. A central component of the manioc production in this region is fire [50], which can escape from cultivation areas and cause large-scale forest fires. Furthermore, overharvesting to feed the increasing food demand has resulted in declines in aquatic [51] and terrestrial [52] wildlife populations across the Amazon.

Deforestation from small-scale agriculture is insignificant in comparison to that of large-scale cattle ranching and soya production, yet the impact of escaped fires can be huge, particularly in the context of climate change and the associated dry conditions. Agroforestry systems have been evaluated in the Amanã SDR (upland) to reduce the impacts of this management, while maintaining local production [15]. In good production practices for *Uarini* manioc flour, the use of pesticides is prohibited and agroecology practices are encouraged [38]. However, the lack of use of fire to clear the fields is still a cultural barrier for this population [15].

Community-based management has seen some of the greatest successes for the recovery of wildlife populations in the Amazon [53, 54]. Following great past social and ecological successes with fisheries management [17], the region is working on developing management programmes for caiman and terrestrial wildlife; the pilot management of wild black caiman *Melanosuchus niger* was undertaken in March 2020, with 28 animals, resulting in 600 kg of meat sold in the town of Tefé.

To develop these management schemes it is necessary to determine sustainable hunting rates through researching fundamental biology such as generation lengths [20], develop safe meat preparation processes, encourage participatory mapping and management area zonation, conduct biological population monitoring [55], and train the riverine people in the practicalities of terrestrial wildlife management, which aims to support local food security [56]. There is a potential for participatory research (*Target 12.A*) with local populations in formulating well-informed decisions for the conservation of natural resources and economic alternatives focused on the conservation of human-natural systems [57].

The riverine people from the Amazon Rainforest used to live sustainable lifestyles. Despite this, an adequate management of the non-organic wastes (*Target 12.5*)—especially those derived from the goods bought in the city’s markets—is a constant challenge due the absence of waste public services in the rural areas.

### SDG 13: Climate action

Goal 13 aims to "Take urgent action to combat climate change and its impacts", with targets such as: (13.1) strengthen resilience and adaptive capacity to climate-related hazards; (13.3) improve education, awareness-raising and human capacity on climate change adaptation; and (13.B) promote mechanisms for raising capacity for effective climate change [2].

Records indicate changes in the Amazon River discharges over the last decades, associated with El Niño and La Niña anomalies and climate change [58]. To anticipate changes in floodplain water levels in the Mamirauá SDR, monitoring has been developed since the early 1990s [59].

Although the riverine residences in the Amazon floodplain are typically well adapted to the water flood pulses (stilt-houses or floating-houses), climate change may increase the water level and the natural erosion of the banks – locally called *terras caídas* ("fallen lands"). These landscape events often force the displacement of settlements to new areas [6]. Stilt-houses are currently about 1.5 m high, and may become higher in the future if those populations choose not to move to other areas. Floating-houses have benefits under water level fluctuation conditions, but also a higher cost to construct [12]. Yet, some technologies applied in the riverine communities of the floodplains are currently adapted to the flood pulses, such as the water supply photovoltaic pumps over floating wooden rafts [32].

The riverine communities are dependent on rivers for their livelihoods; thus, they would be impacted by changes in the rainfall patterns or water levels. Some of these Amazonian communities already report perceptions of climate change; this fact can influence local farming practices, such as their agricultural calendars are based on hydrological seasons [60]. Climate change would also threaten mammal species of the Amazon floodplain, such as the Black-headed squirrel monkey (*Saimiri vanzolinii*), an endemic species which whole distribution is within Mamirauá SDR [61]; this primate will need to adapt to sudden changes in their habitat or migrate to other areas—otherwise could be extinct [62].

Climate action is a world challenge. The Central Amazon rainforest is one of the most protected areas, especially within the reserves, such as Mamirauá and Amanã SDR. The main challenge there is to help people living in those areas to adapt to the changes (*Targets 13.3 and 13.B*).

### SDG 14: Life below water

Goal 14 aims to "Conserve and sustainably use the oceans, seas and marine resources", with targets as: (14.2) sustainably manage and protect ecosystems; (14.4) regulate harvesting and end overfishing, illegal and destructive fishing practices and implement science-based management plans, in order to restore fish stocks; and (14.A) increase scientific knowledge and research capacity [2]. Although Central Amazonia has no borders with the sea, it may be considered a water world due to the Solimões River basin. The influence it exerts over environment, biota and people due to its overwhelming volume (annual water level variation over 12 m) and sediment load is highly significant.

Amazonia has a rich ichthyofauna with around 2,700 fish species listed [63] and new species being described every year, with up to 8,000 estimated. Despite this wealth, less than 10% (200 species) of the listed species are commercialized—with 6 to 12 species representing more than 80% of fish landings to the main ports of the region [64]; this has resulted in overfishing of some species, such as the tambaqui (*Colossoma macropomum*) [51].

The increased urban demand for fish in the 1970s created a fishing pressure on the floodplains and caused a decline of some species stocks [36]. Participatory fisheries management has promoted (since 1999) an increase of wild stocks of *pirarucu* of the order of 68 times in the Central Amazon managed areas. This management provided price control and better access to markets for the fishers, increasing income by almost 270 times over the years [17].

Four of the five genera of Amazonian aquatic mammals (manatees, pink and tucuxi dolphins, giant otters) are under some level of threat [65, 66], with Neotropical otters requiring further research. After the end of the commercial hunting period, manatees (*Trichechus inunguis*) are still culled throughout the region on a subsistence level [67]. Current threats include entanglement of manatee calves in fishing gear, which has generated a number in excess of 100 animals in legal captivity. In the Amanã SDR an innovative community-based rehabilitation center in a natural setting within a protected area was implemented, with the release of twelve animals in the shortest period possible back into the wild. Accidental gillnetting is also the major issue with river dolphins (*Inia* and *Sotalia*) at this time, so awareness and research activities are being implemented. The recovery of giant river otter populations was first documented in the Amanã SDR [68] and reports of conflicts with fishers are currently common in Amazonas state [69]. Contrary to some other species of freshwater wildlife, aquatic mammals’ biological attributes (slow reproduction, small number of offspring, delayed reproduction, long life span) mean that they cannot undergo commercial management; conservation of these species require custodial management with full protection.

Past uncontrolled extensive exploitation also drastically reduced the wild populations of caimans (*Melanosuchus niger* and *Caiman crocodilus*) and river turtles (*Podocnemis sextuberculata, P. unifilis, P. expansa*) in Central Amazon. The prohibition of commercial trade allowed caiman populations to slowly recover [70], enabling harvesting management by riverine communities. The protection of river turtle nests has been taking place in some mid-Solimões riverine beaches for more than 20 years; this was an initiative proposed by the riverine communities to recover their populations, important in their diet [71].

Currently the Central Amazon has many protected areas and management programs, as in the Mamirauá and Amanã SDR. The main challenge now is to maintain this protection and combat illegal and unregulated fishing (*Target 14.4 and 14.6*); either by economic benefits (*14.7*) and/or better access to markets (*14.B*) for those species that support it. The increasing pollution by microplastics is also a challenge in the Amazon region; its presence has already been confirmed in several species of Amazonian fish commonly consumed by the local population [72]. Plastic ingestion was already reported as the probable cause of death of an Amazonian manatee [73].

### SDG 15: Life on land

Goal 15 aims to "Protect, restore and promote sustainable use of terrestrial ecosystems, sustainably manage forests, combat desertification, and halt and reverse land degradation and halt biodiversity loss", with targets such as: (15.2) promote the implementation of sustainable management; (15.5) reduce the degradation of natural habitats, halt the loss of biodiversity, and protect and prevent the extinction of threatened species; and (15.C) combat poaching and trafficking of protected species, including by increasing the capacity of local communities to pursue sustainable livelihood opportunities [2].

The Brazilian Amazon holds a significant biodiversity, and has 28% of its territory designated as protected areas [8]. Amazonia is the Brazilian biome with the most mammal species (399), and 58% are strictly Amazonian. Among these 399 species, there are 146 Chiroptera, 92 Primates, and 18 Carnivora [74].

Eleven primate species from five families live within the boundaries of the Mamirauá SDR [62]. The black-headed squirrel monkey constitutes an example of extreme endemism, with one of the smallest geographic distributions of all Neotropical primates—around 870 km^2^ [61]; thus, it is listed as Vulnerable on national and international threatened species lists [65, 66].

Twenty-one medium and large sized terrestrial mammals were recorded in the area of Mamirauá and Amanã SDR—among the 51 species registered in the entire Amazon; the presence of these species demonstrates the effectiveness of wildlife conservation in large protected areas [75], as nine of these are considered Vulnerable on national and international threatened species lists [65, 66].

One of the mammal species registered in the Amanã SDR (non-flooded upland area) is the paca; despite being considered as Least Concern [65], its populations are decreasing due to the uncontrolled hunting by the riverine people for subsistence consumption [18]. Thus, management programs of this species have been considered, with the participation of local communities, to maintain the livelihoods of the riverine people [20].

Almost 74% of the 92 Brazilian mammals described over the last 20 years are currently considered to be restricted to Brazil, and 53% have restricted ranges; most of them are from Amazonia, and many species have yet to be found [74]. Thus, protecting all this biodiversity is the main challenge in this region—requiring resources and incentives to its conservation and management (*Targets 14.A and 15.B*).

### SDG 16: Peace and justice strong institutions

Goal 16 aims to "Promote peaceful and inclusive societies for sustainable development, provide access to justice for all and build effective, accountable and inclusive institutions at all levels", with targets such as: (16.7) ensure inclusive and participatory decision-making at all levels; (16.10) ensure public access to information, and protect fundamental freedoms; and (16.B) promote and enforce non-discriminatory laws and policies for sustainable development [2].

The SDR concept [9] was a novelty when created (in 1996), since protected areas traditionally remove the people who live within them. Despite the permission for people to live inside the reserve, in the first SDR the residents initially did not have any type of official documentation that regularized their presence in the territory, nor that guaranteed them the right to be there and to explore the resources found there [10].

At the inception of the SDR, some areas supposedly belonged to some self-appointed "owners" of the lands [10]; however, in the Brazilian environmental law, the areas around the rivers are considered as Union Heritage sites. This rural land insecurity is being solved with the Concession of Right of Use, issued for the possession of families in a small portion of the territory where the dwelling is located. Nevertheless, in these SDR there are home domain territories (house, garden, farm) and community domain territories [10]. Thus, these deeds need to be adapted to this reality.

The community participation model was chosen by the residents of the Mamirauá SDR for the management of the reserve, through general assemblies and associations meetings [10]. The members and participants are strengthened through cooperation (local and international) and knowledge training in many areas. The pending land tenure regularization and the management difficulties are still major challenges.

SDR have existed in Brazil for almost 25 years, reaching more than one generation of riverine people in Central Amazon. Nonetheless, fundamental freedoms and policies (*Targets 16.10 and 16.B*) are a continued challenge.

### SDG 17: Partnerships to achieve the goals

Goal 17 aims to "Strengthen the means of implementation and revitalize the global partnership for sustainable development", with targets such as: (17.7) promote the development, transfer, dissemination and diffusion of environmentally sound technologies; (17.16) enhance global partnership to support the achievement of the sustainable development goals; and (17.17) promote effective public, public–private and civil society partnerships [2].

Many social organizations raise funds from national and international sources to invest in research and extension projects in the Central Amazon region. Those projects involve water and sanitation (*SDG 6*), social technologies (*SDG 7*), community-based tourism (*SDG 8*), wildlife conservation (*SDG 1*4), forest management (*SDG 15*), and corresponding areas. These projects act in multisectoral partnerships with universities, institutes, foundations—connecting scientific and technical knowledge with the traditional knowledge of river dwellers and the forest people (Table 1).

The projects developed in Central Amazon are linked, directly or indirectly, with all of the Sustainable Development Goals (SDG); these results have improved the lives of thousands of riverine people that live in the midst of the Amazon Rainforest—while allowing wildlife conservation. The effects are immeasurable; and funding is required to continue this development. Thus, the main challenge is to maintain cooperation with science, technology, innovation, and knowledge sharing (*Target 17.6*) and dissemination of environmentally sound technologies (*17.7*) through multi-stakeholder partnerships (*17.16 and 17.17*).

## Conclusions

The seventeen Sustainable Development Goals have a clear connection with the Sustainable Development Reserves in Central Amazon.

Despite the many achievements conquered over the years, there are many challenges yet to reach; and while striving to achieve the goals from the 2030 Agenda, new challenges will emerge. The current main challenges to reach the Sustainable Development Goals in the Mamirauá and Amanã Sustainable Development Reserves, in Central Amazon, are connecting to the reality of rural areas.

Yet, the Amazon Rainforest is a water world, creating even more complex conditions to spread sustainable development. The Amazonian riverine people have a traditional sustainable livelihood; thus, it is important to find the balance to the improvement of their welfare. Unfortunately, the COVID-19 pandemic will create even greater difficulties to achieve the objectives and targets on time in this region.

## Data Availability

Data sharing not applicable. All data analyzed in this study are included in the cited materials.

## References

[1] World Commission on Environment and Development - WCED, “Report of the World Commission on Environment and Development: Our Common Future,” Nairobi, 1987.

[2] United Nations, “Sustainable Development Goals,” 2015.

[3] Ayres JM. As matas de várzea do Mamirauá. 3rd ed. Belém: Sociedade Civil Mamirauá; 2006.

[4] Junk WJ, Piedade MTF. The Amazon River basin. In: Fraser LH, Keddy PA, editors. The World’s Largest Wetlands: ecology and conservation. New York: Cambridge University Press; 2005. p. 488.

[5] Brasil, Decreto N^o^ 8750, de 9 de maio de 2016. Institui o Conselho Nacional dos Povos e Comunidades Tradicionais. Brasília, DF: Diário Oficial [da] República Federativa do Brasil, 2016.

[6] Moura EAF. Água de beber, água de cozinhar, água de tomar banho: diversidade socioambiental no consumo da água pelos moradores da várzea de Mamirauá, Estado do Amazonas. Cad Saúde Coletiva. 2007;15(4):501–16.

[7] Peralta N, Lima DDM. A comprehensive overview of the domestic economy in Mamirauá and Amanã in 2010. UAKARI. 2014;9(2):33–62. 10.31420/uakari.v9i2.155.

[8] M. do M. A.- MMA. Cadastro Nacional de Unidades de Conservação (CNUC); 2019.

[9] Brasil, Lei N^o^ 9.985, de 18 de julho de 2000. Institui o Sistema Nacional de Unidades de Conservação da Natureza e dá outras providências. Brasília, DF: Diário Oficial [da] República Federativa do Brasil, 2000.

[10] IDSM. Plano de Gestão da Reserva de Desenvolvimento Sustentável Mamirauá. IDSM, Tefé, AM, p. 183; 2014.

[11] do Nascimento ACS, Ed., Plano de Gestão da Reserva de Desenvolvimento Sustentável Amanã. Manaus: SEMA; Sociedade Civil Mamirauá, 2020.

[12] Moura EAF, do Nascimento ACS, Corrêa DSS, Alencar EF, de Sousa IS. Sociodemografia da Reserva de Desenvolvimento Sustentável Mamirauá: 2001–2011. Tefé, AM; 2016.

[13] SIMDE. Sistema de Monitoramento Demográfico e Econômico: Reserva de Desenvolvimento Sustentável Mamirauá. IDSM, Tefé, AM; 2019.

[14] SIMDE. Sistema de Monitoramento Demográfico e Econômico: Reserva de Desenvolvimento Sustentável Amanã. IDSM, Tefé, AM; 2018.

[15] Steward AM, Rognant C, do Brito SV. Roça sem fogo: a visão de agricultores e técnicos sobre uma experiência de manejo na Reserva de Desenvolvimento Sustentável Amanã, Amazonas, Brasil. Biodiversidade Bras 2016;6(2):71–87.

[16] Tregidgo D, Barlow J, Pompeu PS, Parry L. Tough fishing and severe seasonal food insecurity in Amazonian flooded forests. People Nat. 2020;2(2):468–82. 10.1002/pan3.10086.

[17] Gonçalves ACT, da Cunha JBC, da Batista JS. The Amazonian Giant: Sustainable Management of Arapaima (Pirarucu). Tefé, AM: IDSM; 2018.

[18] Valsecchi J, El Bizri HR, Figueira JEC. Caça de subsistência de Cuniculus paca no Médio Solimões, Amazonas, Brasil. Brazilian J Biol. 2014;74(3):560–8. 10.1590/bjb.2014.0098. 10.1590/bjb.2014.0098 25296203

[19] Pereira PM, Valsecchi J, Queiroz H. Spatial patterns of primate hunting in riverine communities in Central Amazonia. Oryx. 2019;53(1):165–73. 10.1017/S0030605317000199.

[20] El Bizri HR, Fa JE, Valsecchi J, Bodmer R, Mayor P. Age at sexual maturity, first parturition and reproductive senescence in wild lowland pacas (Cuniculus paca): Implications for harvest sustainability. Anim Reprod Sci. 2019;205:105–14. 10.1016/j.anireprosci.2019.04.009. 31060921 10.1016/j.anireprosci.2019.04.009

[21] de Medeiros MS, et al. A saúde no contexto de uma reserva de desenvolvimento sustentável: o caso de Mamirauá, na Amazônia Brasileira. Saúde e Soc. 2018;27(1):128–48. 10.1590/s0104-12902018170514.

[22] Moura EAF. Comportamento Reprodutivo Das Mulheres Ribeirinhas Do Amanã. UAKARI. 2005;1(1):31–40. 10.31420/uakari.v1i1.4.

[23] Confalonieri UEC, Margonari C, Quintão AF. Environmental change and the dynamics of parasitic diseases in the Amazon. Acta Trop. 2014;129(1):33–41. 10.1016/j.actatropica.2013.09.013. 24056199 10.1016/j.actatropica.2013.09.013

[24] Hulton G, WHO. Global costs and benefits of drinking-water supply and sanitation interventions to reach the MDG target and universal coverage; 2012, p. 67.

[25] Borges-Pedro JP, Müller P, Nunes AP, Gomes MCRL. Assessment of WASH scenarios in urban and rural schools of a small city in the Brazilian Amazon. Acta Amaz. 2018;48(1):75–82. 10.1590/1809-4392201600263.

[26] Peralta N. Ecotourism as an incentive to biodiversity conservation: the case of Uakari Lodge, Amazonas, Brazil. UAKARI. 2012;8(2):75–94. 10.31420/uakari.v8i2.133.

[27] Peralta N, Cobra L. Turismo de base comunitária: como ocorre a aprendizagem? In: Ozorio RZ, Peralta N, Vieira FS, editors. Lições e reflexões sobre o turismo de base comunitária na Reserva Mamirauá. Tefé, AM: IDSM; 2016. p. 296.

[28] MCTIC. Plano de Ação em Ciência, Tecnologia e Inovação em Extensão Tecnológica para a Inclusão Social. Centro de Gestão e Estudos Estratégicos, Brasília, DF; 2018, p. 28.

[29] Peralta N, Alencar EF. Ecoturismo e Mudança Social na Amazônia Rural: efeitos sobre o papel da mulher e as relações de gênero. Campos. 2008;9(1):109–29. 10.5380/cam.v9i1.

[30] Alencar EF, de Sousa IS. Participação, Cooperação e Empoderamento: a atuação das pescadoras em projetos de gestão de recursos pesqueiros na Reserva de Desenvolvimento Sustentável Mamirauá-AM, Brasil. In: Martínez SA, Hellebrandt L, editors. Mulheres na Atividade Pesqueira no Brasil. Campos dos Goytacazes: EDUENF; 2019. p. 382.

[31] de Sousa JSM, Bezerra NP, Leoni JM, das Oliveira CMM, Amaral MRA. Teçume d’Amazônia: fortalecimento político das mulheres produzindo vitalidade de conhecimentos tradicionais. Amazônica. 2016;8(2):310–40. 10.18542/amazonica.v8i2.5046.

[32] Gomes MCRL, de Nascimento ACS, Corrêa DSS, Brito OS, Moura EAF. Surrounded by sun and water: development of a water supply system for riverine peoples in Amazonia. Rev Tecnol Soc. 2019. 10.3895/rts.v15n35.7912.

[33] Borges Pedro JP, Gomes MCRL, Silva do Nascimento AC. Review of wastewater treatment technologies for application in communities in the Amazonian Varzea. UAKARI. 2011;7(1):59–69. 10.31420/uakari.v7i1.85.

[34] Gomes MCRL, Moura EAF, Borges Pedro JP, Bezerra MM, Brito OS. Sustainability of a sanitation program in flooded areas of the Brazilian Amazon. J Water Sanit Hyg Dev. 2015;5(2):261–70. 10.2166/washdev.2015.123.

[35] Valer LR, Mocelin A, Zilles R, Moura E, Nascimento ACS. Assessment of socioeconomic impacts of access to electricity in Brazilian Amazon: case study in two communities in Mamirauá Reserve. Energy Sustain Dev. 2014;20(1):58–65. 10.1016/j.esd.2014.03.002.

[36] Coelho AA, Peralta NB. ‘Meu patrão é meu dinheiro’: mudanças nas relações comerciais de pequenos produtores rurais do médio Solimões (AM). Rev Econ Política e História Econômica. 2016;35:195–224.

[37] Lee TH, Jan FH. Can community-based tourism contribute to sustainable development? Evidence from residents’ perceptions of the sustainability. Tour Manag. 2019;70:368–80. 10.1016/j.tourman.2018.09.003.

[38] Pires FJ et al. Casa de farinha de mandioca e boas práticas de produção de farinha uarini. Tefé, AM, 2019.

[39] Mendonça AP, Ferraz IDK. Óleo de andiroba: Processo tradicional da extração, uso e aspectos sociais no estado do Amazonas, Brasil. Acta Amaz. 2007;37(3):353–63. 10.1590/S0044-59672007000300006.

[40] Campos-Silva JV, Peres CA. Community-based management induces rapid recovery of a high-value tropical freshwater fishery. Sci Rep. 2016. 10.1038/srep34745. 10.1038/srep34745 PMC5059620 27731319

[41] Denardin VF, Komarcheski R. Farinheiras do Brasil: tradição, cultura e perspectivas da produção familiar de farinha de mandioca. Matinhos: UFPR Litoral; 2015.

[42] da Conceição RM et al. Boas práticas de manipulação do pirarucu. Tefé, AM; 2018.

[43] Penteado IM, et al. Among people and artifacts: actor-network theory and the adoption of solar ice machines in the Brazilian Amazon. Energy Res Soc Sci. 2019;53:1–9. 10.1016/j.erss.2019.02.013.

[44] Pinto ER, Guimarães ACLG, da Silva C. Boas práticas para produção de óleo de andiroba. Tefé, AM; 2019.

[45] Queiroz HL. A reserva de desenvolvimento sustentável Mamirauá. Estud Avançados. 2005;19(54):183–203. 10.1590/s0103-40142005000200011.

[46] Ozorio RZ, Peralta N, Vieira FS. Lições e Reflexões sobre o Turismo de Base Comunitária na Reserva Mamirauá. Tefé, AM: IDSM; 2016.

[47] Amaral E, Torres AC, Peralta N. A avaliação participativa como ferramenta para tomada de decisão em processos de manejo de pirarucu (Arapaima gigas). In: Figueiredo ESA, editor. Biologia, conservação e manejo participativo de pirarucus na Pan-Amazônia. Tefé, AM: IDSM; 2013. p. 278.

[48] Dufour DL, Piperata BA, Murrieta RSS, Wilson WM, Williams DD. Amazonian foods and implications for human biology. Ann Hum Biol. 2016;43(4):330–48. 27337942 10.1080/03014460.2016.1196245

[49] Endo W, Peres CA, Haugaasen T. Flood pulse dynamics affects exploitation of both aquatic and terrestrial prey by Amazonian floodplain settlements. Biol Conserv. 2016;201:129–36. 10.1016/j.biocon.2016.07.006.

[50] Carmenta R, Vermeylen S, Parry L, Barlow J. Shifting cultivation and fire policy: insights from the Brazilian Amazon. Hum Ecol. 2013;41(4):603–14. 10.1007/s10745-013-9600-1.

[51] Tregidgo DJ, Barlow J, Pompeu PS, de Almeida M, Parry L. Rainforest metropolis casts 1,000-km defaunation shadow. Proc Natl Acad Sci. 2017;114(32):8655–9. 10.1073/pnas.1614499114. 28739913 10.1073/pnas.1614499114 PMC5558991

[52] Ripple WJ, et al. Bushmeat hunting and extinction risk to the world’s mammals. R Soc Open Sci. 2016. 10.1098/rsos.160498. 10.1098/rsos.160498 PMC5098989 27853564

[53] Campos-Silva JV, Peres CA, Antunes AP, Valsecchi J, Pezzuti J. Community-based population recovery of overexploited Amazonian wildlife. Perspect Ecol Conserv. 2017;15(4):266–70. 10.1016/j.pecon.2017.08.004.

[54] Castello L, Viana JP, Watkins G, Pinedo-Vasquez M, a Luzadis V. Lessons from integrating fishers of arapaima in small-scale fisheries management at the Mamirauá Reserve, Amazon. Environ Manage. 2009;43(2):197–209. 10.1007/s00267-008-9220-5. 18946698 10.1007/s00267-008-9220-5

[55] Botero-Arias R, Regatieri SA. Construindo as bases para um Sistema de Manejo Participativo dos Jacarés Amazônicos. Tefé, AM; 2013.

[56] Campos-Silva JV, Peres CA, Antunes AP, Valsecchi J, Pezzuti J. The regulation of hunting as a conservation tool of the Amazonian Fauna. Biodiversidade Bras. 2018;8(2):82–8.

[57] Pimenta NC, Barnett AA, Botero-Arias R, Marmontel M. When predators become prey: community-based monitoring of caiman and dolphin hunting for the catfish fishery and the broader implications on Amazonian human-natural systems. Biol Conserv. 2018;222:154–63. 10.1016/j.biocon.2018.04.003.

[58] Gloor M, et al. Intensification of the Amazon hydrological cycle over the last two decades. Geophys Res Lett. 2013;40(9):1729–33. 10.1002/grl.50377.

[59] Ramalho EE, et al. Ciclo hidrológico nos ambientes de várzea da Reserva de Desenvolvimento Sustentável Mamirauá – Médio Rio Solimões, período de 1990 a 2008. UAKARI. 2009;5(1):61–87.

[60] Funatsu BM, Dubreuil V, Racapé A, Debortoli NS, Nasuti S, Le Tourneau FM. Perceptions of climate and climate change by Amazonian communities. Glob Environ Chang. 2019. 10.1016/j.gloenvcha.2019.05.007.

[61] Paim FP, de Sousa e Silva Júnior J, Valsecchi J, Harada ML, de Queiroz HL. Diversity, geographic distribution and conservation of squirrel monkeys, saimiri (Primates, Cebidae), in the floodplain forests of Central Amazon. Int J Primatol. 2013;34(5):1055–76. 10.1007/s10764-013-9714-8.

[62] Paim FP, El Bizri HR, Paglia AP, Queiroz HL. Long-term population monitoring of the threatened and endemic black-headed squirrel monkey (*Saimiri vanzolinii*) shows the importance of protected areas for primate conservation in Amazonia. Am J Primatol. 2019. 10.1002/ajp.22988. 10.1002/ajp.22988 31094012

[63] Dagosta FCP, De Pinna M. The fishes of the Amazon: distribution and biogeographical patterns, with a comprehensive list of species. Bull Am Museum Nat Hist. 2019. 10.1206/0003-0090.431.1.1.

[64] Ruffino ML. A pesca e os recursos pesqueiros na Amazônia brasileira. Manaus: Ibama/ProVárzea; 2004.

[65] IUCN. The IUCN Red List of Threatened Species. Version 2020–1; 2020.

[66] ICMBio. Livro Vermelho da Fauna Brasileira Ameaçada de Extinção. Brasília, DF; 2018.

[67] Marmontel M, de Souza D, Kendall S. *Trichechus inunguis*. The IUCN Red List of Threatened Species 2016: e.T22102A43793736; 2016.

[68] Marmontel M, Calvimontes J, Carvalho O. Rediscovery of Pteronura brasiliensis in the Amanã Sustainable Development Reserve, Amazonas, Brazil. Lat Am J Aquat Mamm. 2015;10(2):147. 10.5597/lajam00207.

[69] Lima DDS, Marmontel M, Bernard E. Conflicts between humans and giant otters (*Pteronura brasiliensis*) in amanã reserve, Brazilian Amazonia. Ambient e Soc. 2014;17(2):127–42. 10.1590/S1414-753X2014000200009.

[70] Marioni B, Botero-Arias R, Fonseca-Junior SF. Local community involvement as a basis for sustainable crocodilian management in protected areas of central Amazonia: problem or solution? Trop Conserv Sci. 2013;6(4):484–92. 10.1177/194008291300600403.

[71] Camillo CS, dos Santos OM, de Sousa IS, de Queiroz HL. Community-based freshwater turtle conservation in Middle Solimões River, AM, Brazil. UAKARI. 2012;8(1):35–44. 10.31420/uakari.v8i1.121.

[72] Ribeiro-Brasil DRG, et al. Contamination of stream fish by plastic waste in the Brazilian Amazon. Environ Pollut. 2020;266: 115241. 10.1016/j.envpol.2020.115241. 32755795 10.1016/j.envpol.2020.115241

[73] Silva AB, Marmontel M. Ingestão de lixo plástico como provável causa mortis de peixe-boi amazônico (Trichechus Inunguis NATTERER, 1883). UAKARI. 2009;1:105–12.

[74] Paglia AP et al. Lista Anotada dos Mamíferos do Brasil/Annotated Checklist of Brazilian Mammals. (2^a^ Edição/2nd Edition); 2012.

[75] Alvarenga GC, Ramalho EE, Baccaro FB, da Rocha DG, Ferreira-Ferreira J, Bobrowiec PED. Spatial patterns of medium and large size mammal assemblages in várzea and terra firme forests, Central Amazonia, Brazil. PLoS ONE. 2018;13(5): e0198120. 10.1371/journal.pone.0198120. 29847606 10.1371/journal.pone.0198120 PMC5976171

